# Using continuous renal replacement therapy to manage patients of shock and acute renal failure

**DOI:** 10.4103/0974-2700.44678

**Published:** 2009

**Authors:** Sachin S Soni, Amit P Nagarik, Gopal Kishan Adikey, Anuradha Raman

**Affiliations:** Department of Nephrology, Mediciti Hospitals, Hyderabad, Andhra Pradesh, India

**Keywords:** Acute renal failure, continuous renal replacement therapy, shock, sepsis

## Abstract

**Background:**

The incidence of acute renal failure (ARF) in the hospital setting is increasing. It portends excessive morbidity and mortality and a considerable burden on hospital resources. Extracorporeal therapies show promise in the management of patients with shock and ARF. It is said that the potential of such therapy goes beyond just providing renal support. The aim of our study was to analyze the clinical setting and outcomes of critically ill ARF patients managed with continuous renal replacement therapy (CRRT).

**Patients and Methods:**

Ours was a retrospective study of 50 patients treated between January 2004 and November 2005. These 50 patients were in clinical shock and had concomitant ARF. All of these patients underwent CVVHDF (continuous veno-venous hemodiafiltration) in the intensive care unit. For the purpose of this study, shock was defined as systolic BP < 100 mm Hg in spite of administration of one or more inotropic agents. SOFA (Sequential Organ Failure Assessment) score before initiation of dialysis support was recorded in all cases. CVVHDF was performed using the Diapact^®^ (Braun) CRRT machine. The vascular access used was as follows: femoral in 32, internal jugular in 8, arteriovenous fistula (AVF) in 4, and subclavian in 6 patients. We used 0.9% or 0.45% (half-normal) saline as a prefilter replacement, with addition of 10% calcium gluconate, magnesium sulphate, sodium bicarbonate, and potassium chloride in separate units, while maintaining careful monitoring of electrolytes. Anticoagulation of the extracorporeal circuit was achieved with systemic heparin in 26 patients; frequent saline flushes were used in the other 24 patients.

**Results:**

Of the 50 patients studied, 29 were males and 21 females (1.4:1). The average age was 52.88 years (range: 20–75 years). Causes of ARF included sepsis in 24 (48%), hemodynamically mediated renal failure (HMRF) in 18 (36%), and acute over chronic kidney disease in 8 (16%) patients. The overall mortality was 74%. The average SOFA score was 14.31. The variables influencing mortality on multivariate analysis were: age [odds ratio (OR):1.65; 95% CI: 1.35 to 1.92; *P* = 0.04], serum creatinine (OR:1.68; 95% CI: 1.44 to 1.86; *P* = 0.03), and serum bicarbonate (OR: 0.76; 95% CI: 0.55 to 0.94; *P* = 0.01). On univariate analysis the SOFA score was found to be a useful predictor of mortality.

**Conclusions:**

Despite advances in treating critically ill patients with newer extracorporeal therapies, mortality is dismally high. Multiorgan dysfunction adversely affects outcome of CRRT. Older age, level of azotemia, and severity of metabolic acidosis are important predictors of adverse outcome.

## INTRODUCTION

Continuous renal replacement therapy (CRRT) has emerged as the dialysis technique of choice for patients of shock with acute renal failure (ARF).[1] It is being increasingly utilized in hemodynamically unstable patients. Whether it is superior to intermittent hemodialysis is still a debatable issue.[2,3] As this therapy is expensive, widespread usage in developing countries like India is not feasible. We report our 2-year experience of treating 50 critically ill cases with CRRT.

## PATIENTS AND METHODS

All critically ill patients undergoing CRRT between January 2004 and December 2006 were retrospectively analyzed. CRRT was advised in critically ill patients with shock and ARF. Shock was defined as systolic BP < 100 mm Hg in spite of administration of one or more inotropic agents. Continuous veno-venous hemodiafiltration (CVVHDF) was performed using the Diapact^®^ CRRT (B. Braun Company).

We used the Diacap Acute (B. Braun) polysulfone filter (effective surface area: 1.2 m^2^ and ultrafiltration coefficient: 42 ml/h/mm Hg). Vascular access included arteriovenous fistula (AVF) or double-lumen 12-French catheter (DLC) inserted into the internal jugular, subclavian, or femoral veins. Hemodiafiltration was accomplished using a blood flow rate of 100–150 ml/min. Commercially available dextrose and potassium-free Hemosol BO (Hospal Division, Gambro Limited) was used as a dialysis solution at a fixed flow rate of 1200 ml/h. A fixed amount of replacement fluid of 400 ml/h by predilution method comprising 0.9% or 0.45% (half-normal) saline was used. Addition of 10% calcium gluconate, magnesium sulphate, 7.5% sodium bicarbonate, and potassium chloride was done, with monitoring of the levels of these electrolytes. Proctoclysis enema (sodium phosphate 10%) given through a nasogastric tube was used for phosphorous replacement as injectable phosphorous replacement solution was unavailable. The ultrafiltration (UF) rate was based on the volume status of the patient as assessed by central venous pressure (CVP) monitoring. Clearance rate per hour was calculated by addition of dialysis solution flow rate and UF rate (clearance rate/hour = 1200 + UF rate/h).

Anticoagulation of the extracorporeal circuit was achieved with systemic heparin, with a target partial thromboplastin time of 65 s in the arterial (prefilter) line. No anticoagulation was used in patients having systemic coagulopathy. SOFA (Sequential Organ Failure Assessment) score[4] before initiation of CRRT was recorded in all cases. ARF was defined as a rise of serum creatinine by 25% over baseline in patients without preexisting renal disease. Acute over chronic kidney disease (CKD) was defined as uncharacteristic change in renal function (increase in serum creatinine by 1 mg/dl or 25% over baseline) developing over days in patients having deranged creatinine (>2 mg/dl) for more than 3 months. Hemodynamically mediated renal failure (HMRF) was defined as ARF developing secondary to shock. Survival or mortality was the main outcome measure of this analysis.

### Statistical analysis

All data was systematically collected and tabulated. All values were expressed as a mean ± standard deviation. Student's t-test was used for analysis of parametric variables and the Mann-Whitney test for nonparametric variables. Mortality was the main outcome measure. Multivariable logistic regression analysis was then conducted to assess the factors influencing mortality. Based on the results of the univariate analysis, the covariates considered were age, male gender, SOFA score, and serum creatinine and serum bicarbonate at CRRT initiation. The SPSS for Windows (Student Version) was used for the statistical analysis. A *P* value of <0.05 was taken as statistically significant.

## RESULTS

**Figure 1:**
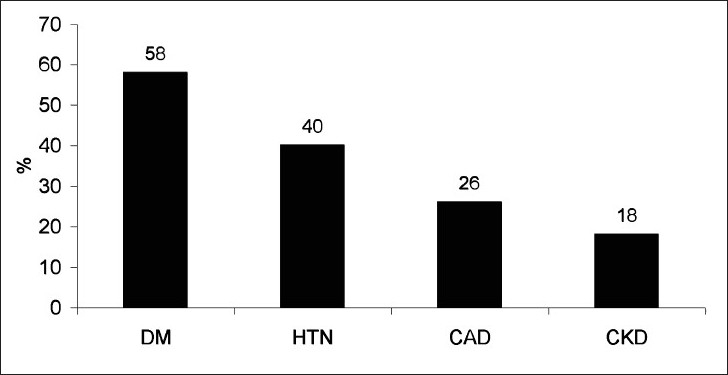
Comorbid illnesses

Of the 50 cases who underwent CRRT, 29 were males and 21 female. Most patients in both genders [Table 1] were elderly (≥60 years of age). Type 2 diabetes mellitus was the most frequent comorbid illness (58%), followed by hypertension (40%), coronary artery disease (26%), and CKD (18%) [Figure 1]. Causes of ARF included sepsis in 48% cases, HMRF in 36% cases, and acute over chronic kidney disease in 16% cases [Table 2]. Source of infection could be identified in 21 (87.5%) cases. Cystitis was the most common infection leading to sepsis, followed by community-acquired pneumonia. Acute myocardial infarction leading to cardiogenic shock was the most common cause of HMRF. Drugs, infection, and contrast media were implicated in acute deterioration of stable CKD [Table 2].

**Table 1:** Age and gender distribution

Age interval (Years)	Male (*n* = 29)	Female (*n* = 21)
20–29	2 (6.8)	4 (19)
30–39	2 (6.8)	0
40–49	5 (17.2)	2 (9.5)
50–59	5 (17.2)	4 (19)
60–69	7 (24.8)	7 (33.5)
>70	8 (27.2)	4 (19)
Mean age	57.06 ± 17.87	54.66 ± 20.59

FIGURES IN PARENTHESIS INDICATE PERCENTAGE

**Table 2:** Etiology of ARF

1. Sepsis	24 (48%)
A. Source of infection identified	21
a. Cystitis	7
b. Community-acquired pneumonia	5
c. Ventilator-associated pneumonia	2
d. Diabetic foot	2
e. Acute pyelonephritis	2
f. Liver abscess	2
g.Cellulitis	1
B. Source of infection not identified	3
2. Hemodynamically mediated renal failure	18 (36%)
A. Cardiogenic shock	10
B. Pulmonary thromboembolism	2
C. Acute pancreatitis	2
D. Postsurgical	2
E. Hypovolemia	2
3. Acute over chronic kidney disease	8 (16%)
A. Drug-induced	3
B. Infection	3
C. Contrast nephropathy	2

The mean duration of CRRT was 37.51 ± 19.37 h with mean clearance rate of 1650 ± 75 ml/h. Femoral vein DLC was the most common vascular access. Heparin anticoagulation was used in 26 (52%) cases, while no anticoagulant was used in 24 (48%) cases. The average life of the Diacap filter was 33.5 ± 18.5 h; the protocol of anticoagulation did not make any significant difference to filter life [Table 3]. The mean SOFA score was 14.31 ± 4.12.

**Table 3:** CVVHDF details

Mean duration (h)	37.51 ± 19.37 (2–168)

Mean clearance rate (ml/h)	1650 ± 75 (1550–1800)

Vascular access	
Femoral vein	32 (64%)
Internal jugular vein	8 (16%)
Subclavian vein	6 (12%)
AVF	4 (08%)

Anticoagulation	
Heparin	26 (52%)
No anticoagulation	24 (48%)

Overall mortality was 74%; it was highest in sepsis (83%) followed by HMRF (77%) and acute over chronic kidney disease (50%). Patients on mechanical ventilator had a mortality of 80% (28 of 35) compared with the mortality of 60% (9 of 15) in the nonventilated group. Univariate analysis suggested older age, higher SOFA score, higher level of azotemia, and lower serum bicarbonate to be associated with higher mortality. Gender, predialysis blood pressure, serum sodium, and serum potassium did not have any influence on mortality [Table 4]. UF rate on CRRT and method of anticoagulation did not influence mortality. On multivariate analysis, SOFA score did not remain a significant predictor of mortality [Table 5].

**Table 4:** Factors influencing mortality by univariate analysis

Factor	Survivors *n* = 13	Non survivors *n* = 37	*P* value
Mean age (Years)	43.33 ± 15.19	56.16 ± 16.3	0.04
Gender			Comparing between A and B, NS
A. Male (*n* = 29)	8 (27.5%)	21 (72.5%)
B. Female (*n* = 21)	5 (23.8%)	16 (76.2%)
Systolic blood pressure (mmHg)	97.33 ± 7.50	92.45 ±12.5	NS
Diastolic blood pressure (mmHg)	61.11 ±7.33	60.96 ± 8.70	NS
SOFA score	11.42 ± 3.12	15.33 ± 4.01	0.03
Serum creatinine (mg/dl)	4.31 ± 1.00	6.93 ± 4.38	0.02
Blood urea (mg/dl)	111.33 ±37.31	134.56 ± 65.5	0.02
Serum sodium (mEq/l)	130.54 ±6.93	130.70 ±7.07	NS
Serum potassium (mEq/l)	5.07 ± 0.86	4.6l ± 1.22	NS
Blood pH	7.28 ± 0.09	7.13 ± 0.09	0.024
Serum bicarbonate (mEq/l)	18.34 ± 15.25	11.96 ± 4.41	0.04
Mechanical ventilation			Comparing between A and B, NS
A. Required (*n*=35)	7 (20%)	28 (80%)	
B. Not required (*n*=15)	6 (40%)	9 (60%)	

NS: NOT SIGNIGICANT.

**Table 5:** Factors influencing mortality by multivariate analysis

Variable	Multivariate analysis		
	
	Odds ratio (OR)	Confidence interval (Cl_95_%)	*P* value
Mean age	1.65	1.35 to 1.92	0.04
Male gender (%)	1.04	0.85 to 2.25	0.89
SOFA score	1.25	0.94 to 1.96	0.16
Serum creatinine	1.68	1.44 to 1.86	0.03
Serum bicarbonate	0.76	0.55 to 0.94	0.01

## DISCUSSION

ARF, also referred to as acute kidney injury (AKI), occurs as a result of ischemic and toxic kidney injury and is a life-threatening complication of sepsis and other syndromes in the intensive care unit (ICU).[5] ARF is an independent predictor of mortality in critically ill cases.[6] The use of continuous therapies was first described three decades ago by Kramer *et al*.[7] In comparison with the Western world, where CRRT is used in 80% of ICU ARF,[8] its use is limited in India. Possible reasons include high cost, technical difficulties, and shortage of adequately trained staff. Most of the patients in our study were elderly, as has also been reported by Turney *et al.*[9] and Mehta *et al*.[10] Sepsis was the most common cause of ARF in our study. A large multinational, multicenter study has identified septic shock as the etiology of ARF in 47.5% cases, which is consistent with our observation.[8]

The mean duration of CRRT was 37.51 ± 19.37 h. Many factors, including clotting of filter, conversion of patients to intermittent hemodialysis after achieving hemodynamic stability, and death of the patients during the therapy determined the duration of treatment. The prohibitive cost did not allow us to replace the filter on a daily basis as has been recommended by some authors.[12] We achieved a mean clearance rate of 1650 ml/h. Taking the average weight of Indian adults to be around 50–60 kg, we achieved a clearance of around 27–33 ml/kg/h. A similar clearance rate has been reported by a large multicenter trial.[13] The clearance achieved in our study is lower than the target clearance of 35 ml/kg/h reported from most Western centers[14]; this is because we avoided weight-based alteration in clearance values and instead followed a fixed prescription protocol to make the procedure less complicated and more user friendly.

The overall mortality in our study was 74%. Many other studies have reported high mortality in critically ill ARF [Table 6]. In our study, older age, azotemia, and metabolic acidosis were predictors of adverse outcome, similar to observations by other authors.[11,18,19] Many studies have tried to address the question of the superiority of one therapy over the other (CRRT *vs* Intermittent hemodialysis) but the results are equivocal. A recently published meta-analysis by Tonelli *et al*. has shown both modalities to be equally effective.(20)

**Table 6:** Mortality in patients treated with CRRT

Author	Type of study	Number of patients	Mortality in patients treated with CRRT	Remarks
Mehta *et al*.[14]	Randomized trial (CRRT *vs* IHD)	84 randomized to CRRT and 82 to IHD	65.5	Unadjusted, in hospital mortality
Vinsonneau *et al*.[13]	Multicenter randomized trial (CRRT *vs* IHD)	360 patients randomized to CRRT or IHD	67	60-day all-cause mortality
Uehlinger *et al*.[17]	Randomized trial (CRRT *vs* IHD)	70 randomized to CRRT and 55 to IHD	47	All-cause mortality
Lobo[15]	Observational study	22 patients treated with CRRT	77	30-day mortality
Our study	Retrospective	50 patients treated with CRRT	74	In-hospital mortality

IHD: INTERMITTENT HEMODIALYSIS.

### Limitations of the study

This study is on critically ill patients of shock and ARF treated with CRRT without the use of any newer intervention. Absence of a control group randomized to either intermittent hemodialysis (IHD) or high-volume peritoneal dialysis is a major limitation of this study. We were also not able to deliver the optimum CRRT dose of 35 ml/kg/h for logistic reasons.

## CONCLUSIONS

Technological advances in the treatment of critically ill renal failure patients are yet to translate into survival benefits. Multiorgan dysfunction adversely affects the outcome of CRRT. Older age, level of azotemia, and severity of metabolic acidosis are important predictors of adverse outcome. Septic shock is the commonest cause of ARF in critically ill patients and is associated with high mortality. In India, the application of the CRRT technique is largely limited to private-sector corporate hospitals. The prohibitive cost and lack of trained staff are the major factors limiting its wider use.
